# An Updated Mini Review of Vitamin D and Obesity: Adipogenesis and Inflammation State

**DOI:** 10.3889/oamjms.2016.103

**Published:** 2016-09-06

**Authors:** Zujaja-Tul-Noor Hamid Mehmood, Dimitrios Papandreou

**Affiliations:** College of Natural and Health Sciences, Zayed University, Abu Dhabi, United Arab Emirates

**Keywords:** 1,25(OH)2D3, obesity, adipocyte, inflammation

## Abstract

Vitamin D related research continues to expand and theorise regarding its involvement in obesity, as both hypovitaminosis D and obesity strike in pandemic proportions. Vitamin D plays an important role in immune system through Vitamin D Receptors (VDR), which are transcription factors located abundantly in the body. Due to this characteristic, it is potentially linked to obesity, which is a state of inflammation involving the release of cytokines from adipose tissue, and exerting stress on other organs in a state of positive energy balance. Research trials in the past couple of years and systematic reviews from SCOPUS and MEDLINE will be discussed. The role of Vitamin D throughout the lifespan (from fetal imprinting until older age), and in various other obesity mediated chronic conditions shall be highlighted. Various mechanisms attributed to the inverse relationship of Vitamin D and obesity are discussed with research gaps identified, particularly the role of adipokines, epigenetics, calcium and type of adipose tissue.

## Introduction

This study reviews articles available on PUBMED, Scopus and Google using the following search keywords: (Vitamin D OR Ergocalciferol OR Cholecalciferol) AND (Leptin/adiponectin/VDR/inflammation/adiposity/body fat/weight). The search was limited to articles in the English language until June 2016. Selected articles were also used to identify further relevant studies. Most relevant research articles were included; usually, review articles and meta-analysis to present an analysed picture of clinical trials regarding relevant information.

Vitamin D is a micronutrient that is categorically non-essential due to the endogenous production in the body with the aid of specific ultraviolet rays, but it has now become an essential component of diet as Vitamin D deficiency now engulfs the world as a pandemic [1]. This idea was established many years ago as Vitamin D inadequacy was observed even among people living closer the equator [2]. Hence, regulations directing fortification of food is either implemented or are under consideration [3]. In the 2011 conference of Institute of Medicine (IOM), the Recommended Dietary Allowance (RDA) (average daily level of intake sufficient to meet the nutrient requirements of nearly all (97%-98%) healthy people) for Vitamin D was set at 600 IU/d for ages 1-70 years and 800 IU/d for ages 71 years and older [4]. According to the Institute of Medicine (IOM) Committee, the scientific evidence supports the key role of Vitamin D in skeletal health and extra-skeletal health; however, the extra-skeletal health outcomes are not yet consistent to establish a cause-and-effect relationship [4]. Doses to treat and/or maintain Vitamin D status are still subjective and research studying the needs across different life spans and conditions continue. Vitamin D status now conjoins many other health conditions after the discovery of Vitamin D binding proteins and their receptors in many tissues [5]. Vitamin D binding receptors (VDR) are transcription factor responsible for extensive biological responses. It is shown to play a role in cell proliferation inhibition, cell maturation, immune system, and possibly colonic, breast and prostate cancer [6]. Vitamin D status is influenced by sun exposure, adiposity, body composition, race/ethnicity, and genetic factors, but these need elucidations through research, as suggested by IOM [6].

Vitamin D deficiency has been historically defined and recently recommended by the Institute of Medicine (IOM) as a 25(OH)D of less than 20 ng/ml. Vitamin D insufficiency has been defined as a 25(OH)D of 21–29 ng/ml. Vitamin D deficiency results in abnormalities in calcium, phosphorus, and bone metabolism. Specifically, vitamin D deficiency causes a decrease in the efficiency of intestinal calcium and phosphorus absorption of dietary calcium and phosphorus, resulting in an increase in PTH levels. Secondary hyperparathyroidism maintains serum calcium in the normal range at the expense of mobilising calcium from the skeleton and increasing phosphorus wasting in the kidneys. The PTH-mediated increase in osteoclastic activity creates local foci of bone weakness and causes a generalised decrease in bone mineral density (BMD), resulting in osteopenia and osteoporosis. Phosphaturia caused by secondary hyperparathyroidism results in a low normal or low serum phosphorus level. This results in an inadequate calcium-phosphorus product, causing a mineralisation defect in the skeleton. This results in an inadequate calcium-phosphorus product, causing a mineralisation defect in the skeleton [7].

A major health issue linked with Vitamin D is the growing obesity rate. World Health Organization states that in 2014 more than 1.9 billion adults were overweight, of which 600 million were obese. According to the Global Burden of Disease report of USA, the potentially avoidable risk factors to rising disease burden included high BMI and physical inactivity for the healthy years lost [8]. Healthy years measure the expected number of years a person of a certain age could live without disability or be free of any activity limitation. Despite increased levels of sufficient physical activity in male and female, only 9 countries experienced a decline in obesity rate in the United States (with statistically insignificant results) compared to increment in the rest of the counties in obesity during 2001 and 2009 [8]. Systematic analysis to study Global, regional and national prevalence of overweight and obesity in children and adults 1980-2013 did not report any significant decline in rate over the past 33 years, but only a slowdown in increase rate of overweight and obesity was observed just in developed countries [9]. This is not satisfactory, as it indicates that an increasing obesity trend endures in other countries. This demands special attention from the public health sector to search and address socio-economic implications in maintaining Vitamin D. To explain the deficiency of this fat-soluble vitamin in people with excessive adipocytes, the following possible mechanisms have been suggested: lower dietary intake; altered behavior that reduces cutaneous synthesis, reduced synthetic capacity, reduced intestinal absorption, altered metabolism, and sequestration in adipose tissue [10,11]. However, extensive research is needed to establish a cause-effect relationship and explain these factors under various conditions, because Vanlint concluded in his review that the evidence for vitamin D affecting fat mass and distribution is not yet compelling, and it is difficult to determine which effects are due to vitamin D itself and which are mediated via calcium when based on evidence from in vitro studies [11].

Finally, this article highlights how these public health issues of Vitamin D with its immune-related properties are associated with obesity, which is a state of low-grade inflammation. Various gaps identified by IOM committee is briefly reviewed, such as, non-skeletal health outcomes, epigenetic role, physiology and pathways of Vitamin D [4]. Researchers have previously reviewed data on this topic, [12, 14, 15], but due to constant addition of knowledge in this area we considered updating. Unlike some previous articles, we have concentrated on discussing Vitamin D and inflammatory properties in obesity, and further supplemented suggested mechanisms with clinical studies.

## Obesity, a state of low-grade inflammation

Ectopic fat storage, due to overflow from adipose tissues upon fat overloading leads to the formation of foam cells from macrophages that engulf fat droplets during transportation or storage. Macrophages phagocytize lipid droplets in weight loss from adipocytes that could describe basic inflammatory nature of adipocytes. Hyperplasia and hypertrophy of adipocytes can cause mitochondrial and endoplasmic stress, which during fat overloading releases additional inflammatory cytokines other than adipokines, attracting more macrophages. During adipocytes hypertrophy TNF-α (Tumor Necrosis Factor- alpha), IL-6 (Interleukin-6), IL-1β (Interleukin-1 Beta), PG-E2 (Prostaglandin E2) expression is induced, adipocytes die and neutrophils, monocytes and T-Cells are persistently activated. CRP is also enhanced in the liver to respond to inflammatory cytokines, amplifying the cytokines pro-inflammatory effect. Hyperplastic adipocytes also induce genes TNF-α, interleukins (IL)-1, IL-6, monocyte chemoattractant protein-1 (MCP-1), and plasminogen activator inhibitor-1 (PAI-1) due to hypoxia from clustered formation, which are distant from the vasculature. Macrophages embedded in adipocytes phagocytize lipid droplets and engulf dead adipocytes that burst from high lipid accumulation, releasing reactive oxygen species and inducing further cellular stress [16]. The unfolded protein response (UPR) to cope with Endoplasmic Reticulum (ER) stress promotes NF-*κ*B (nuclear factor kappa-B) and JNK (c-Jun N-terminal kinases) inflammatory pathways due to increased protein demand such as in hyperglycemia, or during accumulation of misfolded protein. Excessive nutrient influx increases superoxide production and reactive oxygen species by mitochondria. The down-regulation of autophagy in the liver of lipid droplets in hepatocytes associated with obesity leads to accumulation of triglyceride, ER stress and Insulin resistance [17].

Visceral or subcutaneous fat in obesity-related inflammation is still questionable due to variability in results. A study investigating concentrations of pro-inflammatory enzymes presented higher concentration of IL-6 and IL-15 (Interleukin-15) in Subcutaneous Adipose Tissue (SAT) synthesis compared to Visceral Adipose Tissue (VAT). However, obesity was associated with VAT, since IL-6 and IL-15 were significantly more in obese individuals compared to normal-weight ones, whereas, the cytokines difference was not significant between two groups in SAT related cytokines expression [18]. On the other hand, some studies do suggest increased pro-inflammatory cytokines expression in SAT compared to VAT proposing its contribution to meta-inflammation [18, 19]. In conclusion, visceral fat compared to subcutaneous fat may cause metabolic abnormalities by secreting inflammatory adipokines, such as interleukin, tumour necrosis factor-α, macrophage chemoattractant protein-1, and resistin, which induce insulin resistance and diabetes and Vitamin D metabolic abnormalities.

## Role of Vitamin D in obesity and suggested mechanisms

Research to explore the relationship between Vitamin D and obesity gains interest, because studies investigating obesity (a state of low-grade inflammation) and Vitamin D (with its role in immunity) indicate potential links. Many studies report changes in Vitamin D status with BMI changes. A change in serum Vitamin D levels as a function of adiposity/weight loss was noted over 1-2 years [20, 21]. An inverse relationship between Vitamin D and BMI was recognised in Mendelian randomization analysis, [22] and a link with abdominal visceral or subcutaneous adipose tissue was also recognised [23, 24]. In a meta-Analysis of observational studies up to April 2014 in PubMed/Medline, Vitamin D deficiency was prevalent in obese subjects irrespective of age, latitude and cut-offs defining vitamin D deficiency [14].

Another meta-analysis of literature focusing on the last 5 years proposed various mechanisms to discern body weight and Vitamin D relationship, which include: Vitamin D Receptor (VDR) polymorphism shown in transgenic mice and its overexpression in adipocytes that led to fatty acid β-oxidation, lipolysis and reduced energy metabolism; increased parathyroid hormone levels in Vitamin D deficiency that can increase adiposity by influx of calcium into adipocytes promoting lipogenesis; Vitamin D as “essential factor” in leptin depletion which may contribute to increased appetite and obesity in Vitamin D deficient conditions; and outdoor activity, food intake and exercise which can also influence Vitamin D levels as confounding factors [25].

Adipokines relationship with Vitamin D is studied due to their role in obesity. In vitro leptin, secretion by adipose tissue is powerfully inhibited by Vitamin D deficiency [12]. Although clinical trials showed an increase in serum leptin with Vitamin D supplementation, [26, 27] the clinical significance remains to be asserted [12, 28]. A significant effect of Vitamin D supplementation on adiponectin and leptin was not observed in a meta-analysis of 9 Randomized Controlled Trials (RCT) [29]. Serum changes in Vitamin D were significantly associated with plasma leptin levels, independent of plasma adiponectin concentrations. Further larger clinical trials and meta-analysis to effectively review these adipokines, especially focusing on obesity, are needed, as more meta-analysis could not be found.

The dose-response relationship between serum Vitamin D levels have changed and BMI showed a quadratic curve in a research involving various Vitamin D3 doses that suggested rate-limiting mechanism to avoid excessive formation of 1,25-(OH)2D3 (the active metabolite) [30]. The dose-response curves, although parallel, were noted for their difference between the curves, which was approximately 17.5 nmol/L lower for obese subjects compared to normal ones, and approximately 12.5nmol/L lower levels in overweight compared to normal-weight subjects. Extracellular pool size was suggested as the potential factor in this discrepancy rather than fat [29].

Vitamin D-metabolizing enzymes are expressed differently in Adipose tissue as well. There was decreased expression of the 25-hydroxylase CYP2J2 and the 1α-hydroxylase CYP27B1 (which converts 25(OH)D_3_ to the active 1,25(OH)_2_D_3_) in Subcutaneous adipose tissue, and increased expression of CYP24A1 (which inactivates calcitriol binding and activating VDR) after weight loss [30].

Calcium-sensing receptors (CaSR) gene and protein expression were found similar in white adipose tissue of obese and control mice group. Obese group had lower serum vitamin D and amino acid concentrations, and significantly higher serum triglyceride (TG), total cholesterol (TC), low-density lipoprotein-cholesterol (LDL-C), TNF-α, IL-6 and PTH levels, which suggests that Calcium-sensing receptors function through allosteric regulation [31]. CaSR elevates pro-inflammatory cytokines in adipose tissue and decreases cyclic AMP, protein kinase An activity, hormone-sensitive lipase and adipose triglyceride lipase that are key players in the lipolytic pathway [32]. Low Calcium-induced 1,25-di(OH)_2_D_3_ secretion upregulates CaSR expression in adipose tissue, which is followed by an increase in [Ca2+] and reduced lipolysis, and possibly lipogenesis finally yielding fat accumulation in adipocytes. It was shown that higher BMI shows a greater increase in CaSR protein and thus more pro-inflammatory cytokines secreted from obese tissue [33].

## Studies in relationship to Vitamin D and obesity in general population

Obesity in adults is not only of concern for their reduced productivity in life but it may also affect new lives. Observational studies and animal studies now propose and explore the mechanism about how maternal BMI and offspring adiposity from an early age are independently associated, and how in-utero environmental exposures increase susceptibility to obesity and are related to cardiometabolic disorders in later life [34, 35]. An increased risk of prenatal and early postnatal overweight in offspring (1^st^ year of life) was found, which was attenuated by 4 years of age [36]. Vitamin D status during pregnancy could have an epigenetic role since it is not only pivotal in maternal skeletal maintenance and fetal skeletal development, but it could influence fetal “imprinting”, which can affect chronic disease susceptibility soon after birth [37]. Decreased placental expression of VDR in the placenta may be a contributing factor to the pathology of idiopathic FGR (Fetal Growth Restricted)-affected pregnancies [38]. Maternal vitamin D deficiency during pregnancy was associated with impaired lung development in 6-year-old offspring; neurocognitive difficulties at the age of 10, an increased risk of eating disorders in adolescence, and lower peak bone mass at the age of 20 after relevant covariates were adjusted. Randomised controlled trials with long-term follow-up of offspring are required to examine beneficence for offspring and to determine the optimal level of maternal serum 25(OH)D for fetal development [39].

Optimal Vitamin D level is also essential from the adolescent years until the old age that is needed for health benefits. Serum Vitamin D levels decline with puberty onset, and holds a higher risk for obesity, and much greater for Insulin Resistance in pre-pubertal children with suboptimal Vitamin D serum levels [40, 41]. Hypovitaminosis D in overweight or obese adults is registered in many studies, usually accompanied with other health conditions.^13^ Mice on high-fat diet and low-fat diet was treated with calcitriol to demonstrate its effectiveness in reducing obesity-associated renal abnormality. Suggested mechanism was through reduction of cytokines, such as Toll-like Receptors (TLR) that are down-regulated by Vitamin D, hence reducing Interleukin-6 (IL-6) or by preventing abnormal growth of parathyroid hormone (PTH). The lipid droplets were found to be in a degenerative stage in mice fed High-Fat-Diet (HFD) with calcitriol treatment, which showed a causal relationship between calcitriol intake in renal tubules causing structural changes under HFD conditions [42]. A study that included people above 65 years of age suggested an increased risk of vitamin D deficiency in overweight and higher body fat percentages [43]. As previously mentioned, studies also support an inverse relationship between weight loss and Vitamin D serum changes. This is shown to be effective in eliminating obesity-related inflammation since significant reductions in levels of IL-6 were noted with intervention combining Vitamin D3 supplementation and weight-loss program [44]. Low serum 25(OH)D was found to be significantly associated with high serum IL-6 in overweight/obese children and with increased hs-CRP in obese children [45]. 1,25(OH)_2_D_3_ is also found to have a strong inhibitory effect on NFκB signalling in human adipocytes [46]. On the contrary, a meta-analysis conclusive of 13 RCT suggests that Vitamin-D supplementation does not affect inflammatory markers: CRP, TNF-a, IL-6 in overweight or obese subjects [47]. Some studies do not support any link between Vitamin D supplementation and obesity. Supplementation with vitamin D showed no effect on adiposity measures in adults [48]. An increase in serum levels of 25OHD or other inflammatory markers was not observed in overweight and obese youths with 150,000IU supplemented every 3 months, which demands investigation regarding potential dosage and frequency, [49] since another trial with dosage as low as 400IU up till 4800IU daily yielded serum changes when administered for 12 months [50].

## Role of Vitamin D in other obesity mediated diseases

Chronic diseases are usually linked with obesity, which has been further explored in relation to Vitamin D status or to investigate the effectiveness of supplementation in attenuating related symptoms. Visceral obesity has been also found to be related with low levels of Vitamin D [51]. Visceral obesity is also highly correlated with Non-Alcoholic Fatty Liver disease (NAFLD) thus it is expected that Vitamin D is also related with NAFLD. A recent study in adults demonstrated the strong link between vitamin D and NAFLD [52]. The authors examined a total of 1081 adults and concluded that low vitamin D levels were highly associated with NAFLD independent of visceral obesity in subjects with Diabetes or insulin resistance [52]. BMI was also strongly associated with plasma 25- hydroxy Vitamin D, [25(OH)D] and PTH concentrations with possible influence of plasma 25(OH)D in the pathogenesis of hypertriglyceridemia and atherogenic dyslipidemia through inflammation, because the association disappeared when uCRP (ultrasensitive C-Reactive Protein) was introduced as covariable [53]. 25(OH)D low levels and unfavourable lipid patterns have been also found in children [54]. No effect on β-cell function or insulin action in obese non-diabetic adolescents was observed upon administration of Vitamin D3 supplementation [55]. A systematic review provides evidence of the insignificant effect of supplementation with vitamin D on glucose and insulin metabolism in overweight and obese individuals but a positive influence on the serum concentration of 25(OH)D [47]. Serum 25(OH)D level in diabetic patients (Type 2) was found to be inversely correlated with monocyte adhesion to endothelial cells. 1,25(OH)_2_D_3_ suppressed ER stress, [56] and promoted M1-predominant phenotype with lower endothelial adhesion. Vitamin D suppresses both subsets of monocytes, with M1 predominant, however, M1 is involved in advanced plaques compared to M2 in early stages in simple terms [57]. Real paradigms might be more complex and needs further research. A decrease in systolic blood pressure and adiposity in middle-aged subjects after a weight-loss intervention was observed with an increase in plasma 25(OH)D level [58]. Lower inflammatory profile, better insulin sensitivity, higher Vitamin D levels and IGF-1 (Insulin-like Growth Factor-1) in lean mass of obese patients recorded in the study suggest physical activity programs potential to create a better metabolic profile [59]. Hence, special programs to support a lifestyle that incorporate dietary changes and physical activity programs can be used to attain better Vitamin D levels with a range of other health benefits.

In addition, visfatin has been recently found to be associated with Vitamin D levels. Visfatin is a new adipokine involved in several processes. Visfatin plays an important role in inflammatory processes [60]. In a very recent study [61], the authors examined 50 patients with chronic hepatitis with elevated visfatin levels. After administration of vitamin D3 (15,000 IU)/weekly, the patients’ visfatin levels were significantly reduced after a 12, 14, and 48-week period compared with the baseline data. The researchers concluded that Vitamin D supplementation may offer beneficial effects in reducing inflammation in these patients. More studies though are needed to elucidate these optimal effects.

In conclusion, the latest research on Vitamin D deficiency and obesity pandemic supports the role for Vitamin D in prevention and occurrence of obesity. Hence, public health sector needs to address the implying socio-economic aspects influencing Vitamin D status in order to prevent the burden of the disease which the possibly an outcome of Vitamin D deficiency. The adipokines secretion and inflammatory cytokines expression are importantly linked to Vitamin D metabolism. However, the mechanisms need further elucidation, as research is both equivocal and inadequate to establish a direct relationship in some cases. The role of visceral fat is stronger compared to subcutaneous fat in inflammation, related to obesity. The clinical trials identified in this paper usually involve Vitamin D supplementation in attaining sufficient Vitamin D levels, which indicates the need for trials to determine if same effects of Vitamin D can be observed with dietary sources too, as it is imperative in deciding on fortification of food across different socio-economic groups.

## References

[1] Kohlmeier M, Kohlmeier M (2015). Introduction. Nutrient Metabolism: Structures, Functions, and Genes.

[2] Holick MF (2008). The vitamin D deficiency pandemic and consequences for nonskeletal health: mechanisms of action. Mol Aspects Med.

[3] National Institute of Health 2014 Vitamin D Fact Sheet for Health Professionals.

[4] Institute of Medicine (US) (2011). Committee to Review Dietary Reference Intakes for Vitamin D and Calcium. Dietary Reference Intakes for Calcium and Vitamin D.

[5] Ryan JW, Anderson PH, Morris HA (2015). Pleiotropic Activities of Vitamin D Receptors - Adequate Activation for Multiple Health Outcomes. Clin Biochem Rev.

[6] Ross AC, Manson JE, Abrams SA, Aloia JF, Brannon PM, Clinton SK (2011). The 2011 report on dietary reference intakes for calcium and vitamin D from the Institute of Medicine: what clinicians need to know. J Clin Endocrinol Metab.

[7] Hollick MF (2007). Vitamin D Deficiency. N Engl J Med.

[8] Institute for Health Metrics and Evaluation (2013). The State of US Health: Innovations, Insights, and Recommendations from the Global Burden of Disease Study.

[9] Ng M, Fleming T, Robinson M, Thomson B, Graetz N, Margono C (2014). Global, regional, and national prevalence of overweight and obesity in children and adults during 1980–2013: a systematic analysis for the Global Burden of Disease Study 2013. Lancet.

[10] Vanlint S (2013). Vitamin D and obesity. Nutrients.

[11] Hawkins R (2013). Total 25-OH vitamin D concentrations in Chinese, Malays and Indians. Ann Lab Med.

[12] Koszowska A, Nowak J, Dittfeld A, Brończyk-Puzoń A, Kulpok A, Zubelewicz-Szkodzińska B (2014). Obesity, adipose tissue function and the role of vitamin D. Cent Eur J Immunol.

[13] Pourshahidi L (2014). Vitamin D and obesity: current perspectives and future directions. Proc Nutr Soc.

[14] Pereira-Santos M, Costa P, Assis A, Santos C, Santos D (2015). Obesity and vitamin D deficiency: a systematic review and meta-analysis. Obes Rev.

[15] Zuk A, Fitzpatrick T, Rosella L (2016). Effect of Vitamin D3 Supplementation on Inflammatory Markers and Glycemic Measures among Overweight or Obese Adults: A Systematic Review of Randomized Controlled Trials. PLoS One.

[16] Tripathi YB, Pandey V (2012). Obesity and endoplasmic reticulum (ER) stresses. Front Immunol.

[17] Chang YC, Hee SW, Hsieh ML, Jeng YM, Chuang LM (2015). The Role of Organelle Stresses in Diabetes Mellitus and Obesity: Implication for Treatment. Anal Cell Pathol (Amst).

[18] Jonas MI, Kurylowicz A, Bartoszewicz Z, Lisik W, Jonas M, Wierzbicki Z (2015). Interleukins 6 and 15 Levels Are Higher in Subcutaneous Adipose Tissue, but Obesity Is Associated with Their Increased Content in Visceral Fat Depots. Int J Mol Sci.

[19] Spoto B, Di Betta E, Mattace-Raso F, Sijbrands E, Vilardi A, Parlongo RM (2014). Pro- and anti-inflammatory cytokine gene expression in subcutaneous and visceral fat in severe obesity. Nutr Metab Cardiovasc Dis.

[20] Gangloff A, Bergeron J, Pelletier-Beaumont E, Nazare JA, Smith J, Borel AL (2015). Effect of adipose tissue volume loss on circulating 25-hydroxyvitamin D levels: results from a 1-year lifestyle intervention in viscerally obese men. Int J Obes (Lond).

[21] Ceglia L, Nelson J, Ware J, Alysandratos KD, Bray GA, Garganta C (2015). Association between body weight and composition and plasma 25-hydroxyvitamin D level in the Diabetes Prevention Program. Eur J Nutr.

[22] Vimaleswaran KS, Berry DJ, Lu C, Tikkanen E, Pilz S, Hiraki LT (2013). Causal relationship between obesity and vitamin D status: bi-directional Mendelian randomization analysis of multiple cohorts. PLoS Med.

[23] Hannemann A, Thuesen BH, Friedrich N, Volzke H, Steveling A, Ittermann T (2015). Adiposity measures and vitamin D concentrations in Northeast Germany and Denmark. Nutr Metab (Lond).

[24] Kremer R, Campbell PP, Reinhardt T, Gilsanz V (2009). Vitamin D status and its relationship to body fat, final height, and peak bone mass in young women. J Clin Endocrinol Metab.

[25] Yao Y, Zhu L, He L, Duan Y, Liang W, Nie Z (2015). A meta-analysis of the relationship between vitamin D deficiency and obesity. Int J Clin Exp Med.

[26] Ghavamzadeh S, Mobasseri M, Mahdavi R (2014). The Effect of Vitamin D Supplementation on Adiposity, Blood Glycated Hemoglobin, Serum Leptin and Tumor Necrosis Factor-α in Type 2 Diabetic Patients. Int J Prev Med.

[27] Maggi S, Siviero P, Brocco E, Albertin M, Romanato G, Crepaldi G (2013). Vitamin D deficiency, serum leptin and osteoprotegerin levels in older diabetic patients: an input to new research avenues. Acta Diabetol.

[28] Dinca M, Serban M, Sahebkar A, Mikhailidis D, Toth P, Martin S (2016). Does vitamin D supplementation alter plasma adipokines concentrations?A systematic review and meta-analysis of randomized controlled trials. Pharmacol Res.

[29] Gallagher JC, Sai A, Templin I, Thomas Smith L (2012). Dose Response to Vitamin D Supplementation in Postmenopausal WomenA Randomized Trial. Ann Intern Med.

[30] Wamberg L, Christiansen T, Paulsen SK, Fisker S, Rask P, Rejnmark L (2013). Expression of vitamin D-metabolizing enzymes in human adipose tissue -- the effect of obesity and diet-induced weight loss. Int J Obes (Lond).

[31] He Y, Perry B, Bi M, Sun H, Zhao T, Li Y (2013). Allosteric regulation of the calcium-sensing receptor in obese individuals. Int J Mol Med.

[32] Cifuentes M, Fuentes C, Tobar N, Acevedo I, Villalobos E, Hugo E (2012). Calcium sensing receptor activation elevates proinflammatory factor expression in human adipose cells and adipose tissue. Mol Cell Endocrinol.

[33] He YH, Song Y, Liao XL, Wang L, Li G, Alima (2011). The calcium-sensing receptor affects fat accumulation via effects on antilipolytic pathways in adipose tissue of rats fed low-calcium diets. J Nutr.

[34] Patel N, Pasupathy D, Poston L (2015). Determining the consequences of maternal obesity on offspring health. Exp Physiol.

[35] Penfold NC, Ozanne SE (2015). Developmental programming by maternal obesity in 2015: Outcomes, mechanisms, and potential interventions. Horm Behav.

[36] Morales E, Rodriguez A, Valvi D, Iniguez C, Esplugues A, Vioque J (2015). Deficit of vitamin D in pregnancy and growth and overweight in the offspring. Int J Obes (Lond).

[37] Hossein-nezhad A, Holick MF (2013). Vitamin D for Health: A Global Perspective. Mayo Clin Proc.

[38] Murthi P, Yong H, Ngyuen T, Ellery S, Singh H, Rahman R (2016). Role of the Placental Vitamin D Receptor in Modulating Feto-Placental Growth in Fetal Growth Restriction and Preeclampsia-Affected Pregnancies. Front Physiol.

[39] Hart P, Lucas R, Walsh J, Zosky G, Whitehouse A, Zhu K (2014). Vitamin D in Fetal Development: Findings From a Birth Cohort Study. Pediatrics. http://dx.doi.org/10.1542/peds.2014-1860.

[40] Cediel G, Corvalán C, Aguirre C, de Roma-a D, Uauy R (2015). Serum 25-Hydroxyvitamin D associated with indicators of body fat and insulin resistance in prepubertal chilean children. Int J Obes (Lond).

[41] Cediel G, Corvalan C, Lopez de Romana D, Mericq V, Uauy R (2016). Prepubertal Adiposity, Vitamin D Status, and Insulin Resistance. Pediatrics.

[42] Alkharfy KM, Ahmed M, Yakout SM, Al-Daghri NM (2015). Effects of calcitriol on structural changes of kidney in C57BL/6J mouse model. Int J Clin Exp Med.

[43] Oliai AS, van Dijk SC, Ham AC, Brouwer-Brolsma EM, Enneman AW, Sohl E (2015). BMI and Body Fat Mass Is Inversely Associated with Vitamin D Levels in Older Individuals. J Nutr Health Aging.

[44] Duggan C, de Dieu Tapsoba J, Mason C, Imayama I, Korde L, Wang CY (2015). Effect of Vitamin D3 Supplementation in Combination with Weight Loss on Inflammatory Biomarkers in Postmenopausal Women: A Randomized Controlled Trial. Cancer Prev Res (Phila). http://dx.doi.org/10.1158/1940-6207.CAPR-14-0449.

[45] Rodriguez-Rodriguez E, Aparicio A, Andres P, Ortega RM (2014). Moderate vitamin D deficiency and inflammation related markers in overweight/obese schoolchildren. Int J Vitam Nutr Res.

[46] Ding C, Wilding JP, Bing C (2013). 1,25-dihydroxyvitamin D3 protects against macrophage-induced activation of NFkappaB and MAPK signalling and chemokine release in human adipocytes. PLoS One.

[47] Jamka M, Wozniewicz M, Jeszka J, Mardas M, Bogdanski P, Stelmach-Mardas M (2015). The effect of vitamin D supplementation on insulin and glucose metabolism in overweight and obese individuals: systematic review with meta-analysis. Sci Rep.

[48] Chandler PD, Wang L, Zhang X, Sesso HD, Moorthy MV, Obi O (2015). Effect of vitamin D supplementation alone or with calcium on adiposity measures: a systematic review and meta-analysis of randomized controlled trials. Nutr Rev. http://dx.doi.org/10.1093/nutrit/nuv012.

[49] Shah S, Wilson DM, Bachrach LK (2015). Large Doses of Vitamin D Fail to Increase 25-Hydroxyvitamin D Levels or to Alter Cardiovascular Risk Factors in Obese Adolescents: A Pilot Study. J Adolesc Health.

[50] Gallagher JC, Peacock M, Yalamanchili V, Smith LM (2013). Effects of vitamin D supplementation in older African American women. J Clin Endocrinol Metab.

[51] Cheng S, Massaro JM, Fox CS, Larson MG, Keyes MJ (2010). Adiposity, cardiometabolic risk, and vitamin D status: the Framingham Heart Study. Diabetes. http://dx.doi.org/10.2337/db09-1011.

[52] Seo Ji A, Eun CR, Cho H, Lee SK, You HG, Kim SG (2013). Low vitamin D status is associated with nonalcoholic Fatty liver disease independent of visceral obesity in Korean adults. PLoS One.

[53] Guasch A, Bullo M, Rabassa A, Bonada A, Del Castillo D, Sabench F (2012). Plasma vitamin D and parathormone are associated with obesity and atherogenic dyslipidemia: a cross-sectional study. Cardiovasc Diabetol. http://dx.doi.org/10.1186/1475-2840-11-149.

[54] Rusconi RE, De Cosmi V, Gianluca G, Giavoli C, Agostoni C (2015). Vitamin D insufficiency in obese children and relation with lipid profile. Int J Food Sci Nutr.

[55] Javed A, Vella A, Balagopal PB, Fischer PR, Weaver AL, Piccinini F (2015). Cholecalciferol supplementation does not influence beta-cell function and insulin action in obese adolescents: a prospective double-blind randomized trial. J Nutr.

[56] Riek AE, Oh J, Darwech I, Moynihan CE, Bruchas RR, Bernal-Mizrachi C (2014). 25(OH) vitamin D suppresses macrophage adhesion and migration by downregulation of ER stress and scavenger receptor A1 in type 2 diabetes. J Steroid Biochem Mol Biol.

[57] Riek AE, Oh J, Sprague JE, Timpson A, de las Fuentes L, Bernal-Mizrachi L (2012). Vitamin D suppression of endoplasmic reticulum stress promotes an antiatherogenic monocyte/macrophage phenotype in type 2 diabetic patients. J Biol Chem.

[58] Ibero-Baraibar I, Navas-Carretero S, Abete I, Martinez JA, Zulet MA (2015). Increases in plasma 25(OH)D levels are related to improvements in body composition and blood pressure in middle-aged subjects after a weight loss intervention: Longitudinal study. Clin Nutr.

[59] Fornari R, Francomano D, Greco EA, Marocco C, Lubrano C, Wannenes F (2015). Lean mass in obese adult subjects correlates with higher levels of vitamin D insulin sensitivity and lower inflammation. J Endocrinol Invest.

[60] AL-Suhaimi EA, Shehzad A (2013). Leptin, resistin and visfatin: the missing link between endocrine metabolic disorders and immunity. Eur J Med Res.

[61] Sabry D, Al-Ghussein M, Hamdy G, Abul-Fotouh A, Motawi T, El Kazaz A (2015). Effect of vitamin D therapy on interleukin-6, visfatin, and hyaluronic acid levels in chronic hepatitis C Egyptian patients. Clin Risk Manag.

